# Use of the BOADICEA Web Application in clinical practice: appraisals by clinicians from various countries

**DOI:** 10.1007/s10689-017-0014-x

**Published:** 2017-06-16

**Authors:** Anne Brédart, Jean-Luc Kop, Antonis C. Antoniou, Alex P. Cunningham, Antoine De Pauw, Marc Tischkowitz, Hans Ehrencrona, Sylvie Dolbeault, Léonore Robieux, Kerstin Rhiem, Douglas F. Easton, Peter Devilee, Dominique Stoppa-Lyonnet, Rita Schmutlzer

**Affiliations:** 1 Institut Curie, Supportive Care Department, Psycho-oncology Unit, 26 rue d’Ulm, 75005 Paris Cedex 05, France; 2 University Paris Descartes, 71 avenue Edouard Vaillant, 92774 Boulogne-Billancourt, France; 3 Université de Lorraine, Inter-Psy, 3 Place Godefroy de Bouillon, 54015 Nancy Cedex, France; 4 Centre for Cancer Genetic Epidemiology, Department of Public Health and Primary Care, University of Cambridge, Worts Causeway, Cambridge, CB1 8RN UK; 5 Institut Curie, Cancer Genetic Clinic, 26 rue d’Ulm, 75005 Paris Cedex 05, France; 6 Department of Medical Genetics, University of Cambridge, Level 6 Addenbrooke’s Treatment Centre Cambridge Biomedical Campus, Box 238, Cambridge, CB2 0QQ UK; 7 Department of Clinical Genetics, Laboratory Medicine, Office for Medical Services and Department of Clinical Genetics, Lund University, Universitetssjukhuset, 221 85 Lund, Sweden; 8 Familial Breast and Ovarian Cancer Centre, Cologne University Hospital and Faculty of Medicine, Kerpener Str. 34 I, 50931 Cologne, Germany; 9 Department of Human Genetics, Department of Pathology, Leiden University Medical Centre, S4-P, P.O. Box 9600, 2300 RC Leiden, The Netherlands; 10 CESP, University Paris-Sud, UVSQ, INSERM, University Paris-Saclay, 16 Avenue Paul Vaillant-Couturier, 94807 Villejuif Cedex, France

**Keywords:** Breast cancer, Risk prediction model, Tool, Appraisal, Clinical practice, Survey

## Abstract

The ‘BOADICEA’ Web Application (BWA) used to assess breast cancer risk, is currently being further developed, to integrate additional genetic and non-genetic factors. We surveyed clinicians’ perceived acceptability of the existing BWA v3. An online survey was conducted through the BOADICEA website, and the British, Dutch, French and Swedish genetics societies. Cross-sectional data from 443 participants who provided at least 50% responses were analysed. Respondents varied in age and, clinical seniority, but mainly comprised women (77%) and genetics professionals (82%). Some expressed negative opinions about the scientific validity of BOADICEA (9%) and BWA v3 risk presentations (7–9%). Data entry time (62%), clinical utility (22%) and ease of communicating BWA v3 risks (13–17%) received additional negative appraisals. In multivariate analyses, controlling for gender and country, data entry time was perceived as longer by genetic counsellors than clinical geneticists (*p* < 0.05). Respondents who (1) considered hormonal BC risk factors as more important (*p* < 0.01), and (2) communicated numerical risk estimates more frequently (*p* < 0.001), judged BWA v3 of lower clinical utility. Respondents who carried out less frequent clinical activity (*p* < 0.01) and respondents with ‘11 to 15 years’ seniority (*p* < 0.01) had less favourable opinions of BWA v3 risk presentations. Seniority of ‘6 to 10 years’ (*p* < 0.05) and more frequent numerical risk communication (*p* < 0.05) were associated with higher fear of communicating the BWA v3 risks to patients. The level of genetics training did not affect opinions. Further development of BWA should consider technological, genetics service delivery and training initiatives.

**Electronic supplementary material:**

The online version of this article (doi:10.1007/s10689-017-0014-x) contains supplementary material, which is available to authorized users.

## Introduction

Breast cancer (BC) is a major public health problem for women with almost 1.7 million new BC diagnoses estimated worldwide in 2012 [1]. Among these BC patients, 10 to 20% present with a BC family history, and two decades ago, *BRCA1* and *BRCA2* were identified as major BC susceptibility genes [2]. Recently, additional genetic factors have been identified, including rare variants in genes other than *BRCA1* and *BRCA2* associated with “moderate” to “high” risk of BC, and common genetic variants which individually are associated with low BC risk [3].

Next-generation sequencing, whole-exome, whole-genome and gene panel sequencing are recent technological advances that allow for more genes to be simultaneously sequenced than *BRCA1* and *BRCA2* alone, at a reduced cost and a faster turn-around. These panels include a variable number of genes [4–6] and clear evidence of association with breast cancer is currently available for eleven genes (i.e., *BRCA1*, *BRCA2*, *TP53*, *PALB2*, *PTEN*, *CHEK2*, *ATM*, *NF1*, *PTEN*, *STK11*, *CDH1*) [7], supporting the use of this information in clinical genetics services.

This study was performed as part of the BRIDGES research program [8] that aims to implement comprehensive genetic testing into BC risk assessment. The latter will be achieved through further development of the ‘breast and ovarian analysis of disease incidence and carrier estimation algorithm’ (BOADICEA) which presently computes *BRCA1* and *BRCA2* mutation carrier probabilities and future risks of developing breast and ovarian cancer on the basis of explicit disease inheritance patterns, family history and genetic testing information [9–13].

The BOADICEA model and BOADICEA Web Application version 3 (BWA v3, http://ccge.medschl.cam.ac.uk/boadicea/boadicea-web-application/) also allow for demographic factors and tumour pathology information such as the oestrogen and progesterone receptors, HER2, CK5/6, CK14 status of BC in family member(s) to be taken into account [12].

BWA v3 was released for general use to the healthcare community in February 2014, and had been in widespread use for over 2 years at the time this survey was conducted. As a result, the survey respondents’ views principally reflect their experience of using BWA v3 in clinical practice. In April 2016 (1 month before the start of the survey), BWA v4 was released (currently under beta-testing) which included the effects of truncating mutations in *PALB2, CHEK2 and ATM* [13]. Within BRIDGES, the BOADICEA model will be extended to include additional known breast cancer susceptibility genes.

The clinical acceptability of the BOADICEA model and BWA v3 need to be evaluated to inform further development. In practice, several factors have been shown to hamper the clinical implementation of BC risk decision support tools [14] including: (1) logistic barriers (e.g. time required to use BWA v3), (2) clinical barriers (e.g. beliefs in personal clinical intuition against trust in the tool’s usefulness [15]), and (3) educative barriers (e.g. skills needed to understand numerical risk estimates and to communicate them to patients [16]).

Although reservations have been expressed with regard to the use of the BWA in clinical practice, to our knowledge, no large quantitative report is yet available. Such data would inform the further development of this tool and help to address the needs of clinical users.

The present study addressed the following research questions:

1. How acceptable for clinical use is the BWA v3 in terms of clinicians’ assessment of data entry timing, clinical utility, presentation and ease of communicating cancer risks?
2. To what extent are these considerations affected by the user’s profession, weekly clinical genetic activity level, clinical seniority, specific genetics training attendance, importance attributed to BC modifying risk factors and tendency to communicate risk numerically?

## Methods

This was a cross-sectional study.

The overall survey content and example of questions are provided in Table 1. The survey included four sections addressing: (1) practice in genetic counselling and testing for cancer predisposition (14 questions); (2) importance attributed to BC risk factors, including modifying BC risk factors; BWA v3 frequency of use and data entry time; ways of communicating risk (i.e., in relative, absolute, absolute over 5, 10 or 15 year forms) (26 questions); (3) BWA v3 aspects’ assessment (13 questions); and (4) socio-demographic and professional background (8 questions) (Supplementary material).

**Table 1:** Survey items and measures

Survey section and examples of items	Number of items	Scoring and psychometric information
Section 1. Practice of genetic counselling and testing for cancer predisposition	14	
Counselling for breast or ovarian cancer genetic predisposition		5-point scale: none to more than 20 patients per week An overall indicator of clinical genetic activity level was created based on 6 items after multiple correspondence analyses (MCA) [17] A yes/no item asked whether the respondent was a clinical geneticist or genetic counsellor (No = another professional)
Counselling for other cancer genetic predisposition		
Ordering a genetic test in case of breast or ovarian cancer predisposition		
Disclosing cancer genetic test results		
Ordering a genetic test for breast cancer treatment decision making		
Ordering a genetic test for ovarian cancer treatment decision making		
Section 2. Breast cancer risk factors perceived importance and use of risk prediction models	26	
Modifying risk factors		
1. Age at first menstrual period		5-point: Least to most important Three factors were evidenced after principal component analysis (PCA) [18] allowing for computing continuous variables related to the importance given to reproductive (items 1, 2, 4); lifestyle (items 4, 5, 8) and hormonal factors (items 6, 7). Item 3 (BMI) was considered a single item
2. Age at menopause		
3. Body mass index (BMI)		
4. Child bearing at younger age		
5. Alcohol consumption		
6. Smoking		
7. Oral contraception		
8. Hormone replacement therapy		
9. Physical exercise		
Perceived BOADICEA use frequency and data entry time		5-point: never to always. Time in minutes
Frequency of numerical risk communication Figures in relative, absolute or absolute over 5, 10, 15 years lifetime risks		5-point: never to very often
Section 3. BOADICEA model and web-based tool appraisals	13	
1. I think that my clinical judgement is as good as or better than the estimates provided by this tool		5-point: strongly agree (negative appraisal) to strongly disagree (positive appraisal) Three factors were evidenced after principal component analysis (PCA) including items 1 to 3 (factor 1), 4 to 7 (factor 2), 8 and 9 (factor 3) However only the two latter factors presented adequate reliability estimates, allowing for computing continuous attitude variables on perceived risk presentation comprehension (Cronbach’s α = 0.82) and risk communication ease using BOADICEA tool (Cronbach’s α = 0.87) A higher score reflect a positive appraisal of BOADICEA tool
2. I think the tool is not sufficiently scientifically supported or validated for use in my practice		
3. I think data entry takes too much time using this tool		
4. I have not enough skills/training to understand the estimates provided by this tool		
5. I think the probabilities and percentages provided in the output tables are difficult to understand		
6. I think the graphs showing risk curves are difficult to understand		
7. I think the timeframe of the risk estimates is unclear		
8. I fear of upsetting patients using this tool with them		
9. I fear that patients misunderstand their risks using this tool with them		
BOADICEA estimates perceived clinical validity		
BOADICEA numerical and graphical results change clinical intention* against risk reduction mastectomy*		4-point: never to very often A one factor was evidenced from principal component analysis (PCA) allowing for a continuous variable measuring perceived BOADICEA estimates clinical utility (Cronbach’s α = 0.78). A higher score represents a higher perceived clinical utility
BOADICEA numerical and graphical results change clinical intention* against risk reduction salpingo-oophorectomy *		
Section 4. Socio-demographic and professional background	8	
Age, gender, country of clinical practice		Country was categorized based on the number of respondents by country to have at least 6% respondents by category
Declared medical profession		Nine options and other. Due to professional category size, only clinical geneticists, genetic counsellors and specialists (gynaecologist/obstetrician, radiologist, oncology surgeon, breast specialists…) were compared
Clinical seniority		5-point: 1–5 years to 21 years of more experience in providing patient care, does not apply
Declared specific genetic training		Yes/no

### Survey development

The questionnaire was developed in line with BRIDGES’ objectives, which specified the assessment of the BOADICEA model and BWA v3 acceptability in clinical practice. Two questionnaires were identified from published studies addressing similar objectives: a study-specific questionnaire developed to assess clinicians’ views of BC risk prediction models [19] and two validated instruments to assess clinicians’ perceived usability and satisfaction with a decision aid for women carrying a *BRCA1* or *BRCA2* mutation [20]. These were adapted, thus providing a 9-item questionnaire addressing the perceived clinical utility of BWA v3, risk estimates presentation and ease of communication [20] and a four-item questionnaire designed to assess how BWA v3 risk estimates might impact clinical judgement in practice [19] (see Table 3 in the “Results” section).

A preliminary version of the overall survey was designed following survey methods recommendations [21, 22]. It was pilot-tested with clinical geneticists, genetic counsellors, gynaecologists, psycho-oncologists, a radiotherapist and a methodologist (n = 21).

### Online participants’ recruitment

An online survey involving one reminder was conducted using the LimeSurvey application (http://www.limesurvey.org) [23] during May to September 2016.

First, the survey targeted clinicians who were among the potential 7500 individuals who registered to use the BWA since 2007. Second, in order to reach potential non-users of the BWA from genetic clinics, BRIDGES investigators were solicited to contact members of their National Genetics Societies (NGS) who were also invited to complete the survey. A total of 225, 170, 37 and 32 individuals were contacted from the British, French, Dutch and Swedish NGS, respectively.

### Data analysis

**Fig. 1:**
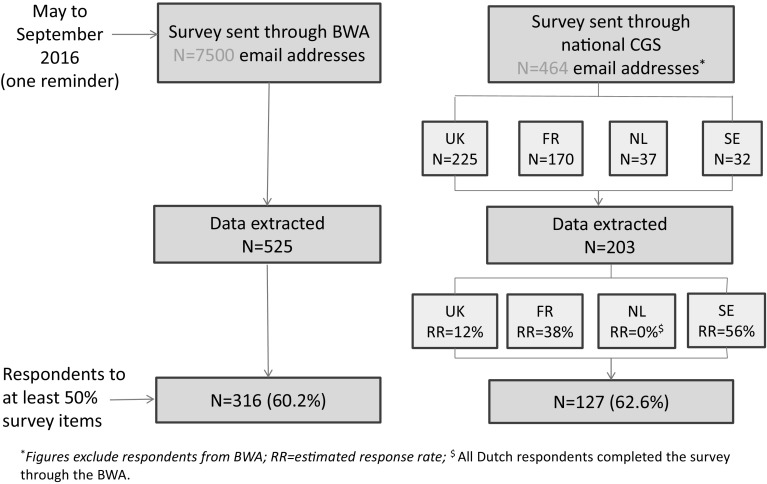
Respondents’ sample

As shown in Fig. 1, 525 and 203 respondents’ data were extracted from the BWA and NGS survey sources respectively. The response rate obtained from the BWA survey source could not be estimated as the survey was sent to registered individuals who might no longer use the tool, and tracking BWA v3 use is not legally permitted. Among respondents contacted through NGS, the response rate was 43.7% (203 respondents out of 464 NGS members, excluding those who responded through the BWA website).

As indicated in Table 1, we developed indicators of genetic clinical activity level, importance attributed to modifying BC risk factors (i.e., reproductive, lifestyle, hormonal and body mass index), and for the BWA v3 appraisals. Higher scores on the three BWA v3 appraisal variables reflect favourable opinions.

Responses were reported in frequencies (percentages). Associations between BWA v3 data entry time and frequency of use (occasionally, regularly, always) were assessed using χ^2^ tests.

We performed multivariate linear regression analyses [24] for the continuous variables including the BWA v3 perceived data entry timing, estimates of clinical utility, risk presentation and ease of risk communication. Professional background characteristics, importance attributed to modifying BC risk factors and mode of numerical risk communication were explored as potential explanatory variables. We controlled for clinicians’ gender and country of practice. Age was significantly correlated to respondents’ clinical seniority so to maintain parsimony it was not included as an explanatory variable. Correlation coefficients between risk communication modes (i.e., using relative, absolute, absolute over 5, 10 or 15 year figures) ranged from 0.15 to 0.36. As the absolute figure mode presented the highest correlation with BWA v3 use frequency (*r* = 0.20), only this mode was included as an explanatory variable.

Statistical analyses were performed with R software version 3.3.1 [25].

## Results

Overall, 443 respondents completed at least 50% of the survey, comprising 316 (71.3%) and 127 (28.7%) through the BWA and NGS survey, respectively (Fig. 1). Successive survey sections were gradually less frequently completed, and as a consequence, there were fewer data for the last section addressing respondents’ socio-demographic and professional characteristics (N = 394).

### Sample characteristics

As shown in Table 2, a wide range of countries were represented among respondents, including France (22%), United Kingdom (20%), Western European countries (other than France and Germany) (13%), USA (12%), Australia and Germany (8%), Canada, Southern European and other countries (6%). Respondents also varied by age and years of clinical experience but were mainly female (77%) and most declared completion of genetics training (e.g., master’s degree in genetic counselling, clinical genetics, residency/internship experience, conference attendance) (70%).

**Table 2:** Sample characteristics (N = 394)

Respondents	N (%)
Age	
20–39	198 (50)
40–49	82 (21)
>50	114 (29)
Gender	
Female	305 (78)
Country	
Australia	30 (8)
Canada	23 (6)
France	86 (22)
Germany	30 (8)
Southern Europe (e.g., Italy, Spain)	25 (6)
United Kingdom	77 (20)
United States	46 (12)
Other Western Europe (e.g., Belgium, Netherlands, Sweden)	51 (13)
Others (e.g., Argentina, Estonia, India, Israel, Taiwan)	25 (6)
Health profession	
Clinical geneticists	115 (29)
Genetic counsellors/nurses	209 (53)
Specialists (e.g., gynaecologists/obstetricians, oncologists, surgeons, breast specialists…)	48 (12)
Others [e.g., general practitioners, junior doctors, genetic lab, (bio) statisticians…]	22 (6)
Seniority/clinical experience	
<11 years	194 (49)
11–15 years	62 (16)
>15 years	130 (33)
Training relevant to genetics	
Yes	274 (70)

### BOADICEA Web Application appraisals and data entry time

Less than 10% of respondents expressed negative opinions on 5 out of 9 items addressing BWA v3 aspects. Some expressed negative opinions about the scientific validity of BOADICEA (9%) and BWA v3 risk presentations (7–9%). Data entry time (62%) and estimates of clinical utility (22%) received additional negative appraisals. Moreover, a significant minority of respondents expressed fear of patients’ misunderstanding (17%) and fear of upsetting patients (13%) using BWA v3 in clinical practice.

About half of respondents indicated that the risk estimates would not change their clinical judgement about breast or ovarian cancer risk management (Table 3).

**Table 3:** BOADICEA appraisal items

	Strongly agree/agree N (%)
I think that my clinical judgment is as good or better than the estimates provided by this tool	88 (22)
I think the tool is not sufficiently scientifically supported or validated for use in my practice	36 (9)
I think data entry takes too much time using this tool	248 (62)
I have not enough skills/training to understand the estimates provided by this tool	36 (9)
I think the probabilities/percentages provided by the output tables are difficult to understand	26 (7)
I think the graphs showing risk curves are difficult to understand	29 (7)
I think the timeframe of the risk estimates is unclear	29 (7)
I fear of upsetting patients using this tool with them	52 (13)
I fear that patients misunderstand their risks using this tool with them	68 (17)
	Never
You think that the patient would be eligible for risk reduction mastectomy *but after looking at BOADICEA numerical and graphical results you think that she is* ***not*** *eligible for risk reduction mastectomy anymore*	155 (40)
You think patient would **NOT** be eligible for risk reduction mastectomy *but after looking at BOADICEA numerical and graphical results you think that she is eligible for risk reduction mastectomy*	166 (43)
You think that the patient would be eligible for risk reduction salpingo-oophorectomy *but after looking at BOADICEA numerical and graphical results you think that she is* ***not*** *eligible for risk reduction salpingo-oophorectomy anymore*	213 (55)
You think patient would **NOT** be eligible for risk reduction salpingo-oophorectomy *but after looking at BOADICEA numerical and graphical results you think that she is eligible for risk reduction salpingo-oophorectomy*	226 (58)

Number of respondents range from 390–400

The overall mean time (standard deviation) taken for data entry using BWA v3 was 15.5 (10.9) minutes (data not shown). The data entry time was not associated with frequency of use, or with whether the respondent was a clinical geneticist, a genetic counsellor or another clinician (Table 4).

**Table 4:** Data entry time by BOADICEA use frequency

	Occasionally		Regularly		Always	
	Mean (SD)	N (%)	Mean (SD)	N (%)	Mean (SD)	N (%)
Clinical geneticists	18.3 (13.7)	24 (22)	16.5 (11.9)	50 (45)	13.9 (10.7)	36 (33)
Genetic counsellors	14.7 (7.6)	47 (23)	16.1 (9.8)	85 (42)	14.1 (8.0)	69 (34)
Others	19.7 (24.1)	23 (35)	14.5 (6.7)	17 (26)	15.2 (8.2)	25 (38)

No significant difference according to use frequency, being geneticists (N = 110) and genetic counsellors (N = 201) versus other clinicians (N = 65) or their interaction. N = 376 as in these analyses respondents who reported not knowing or not using BOADICEA were excluded

### Predictors of BOADICEA Web Application appraisals

In multivariate analyses, data entry time using BWA v3 was perceived as longer by genetic counsellors than clinical geneticists (*p* < 0.05). BOADICEA clinical utility was perceived less favourably with increasing importance given to hormonal BC risk factors (*p* < 0.01) and with the fact it displays numerical risk estimates (absolute figure) (*p* < 0.001) (Table 5).

**Table 5:** Predictors of BOADICEA use perceived data entry time, BOADICEA perceived clinical utility, risk presentation comprehension and communication ease

	Perceived data entry time^a^	Perceived clinical utility^b^	Perceived risk presentation comprehension^c^	Perceived risk communication ease^d^
	*β*	*β*	*β*	*β*
Gender (male)	−0.15**	−0.11	−0.07	0.002
Country (Australia vs. United Kingdom)	0.14*	0.13*	0.05	0.07
Country (Canada vs. United Kingdom)	0.07	0.06	−0.01	0.05
Country (France vs. United Kingdom)	0.18*	−0.07	−0.11	−0.29***
Country (Germany vs. United Kingdom)	0.22***	0.04	−0.03	0.09
Country (others vs. United Kingdom)	0.11	−0.03	0.07	0.09
Country (South European vs. United Kingdom)	0.16**	0.07	0.07	−0.05
Country (United States vs. United Kingdom)	0.001	0.19**	0.05	0.18**
Country (Other West European vs. United Kingdom)	0.29**	0.07	0.02	0.03
Medical profession (genetic counsellors vs. clinical geneticists)	0.15*	0.01	−0.02	−0.01
Medical profession (specialists vs. clinical geneticists)	0.01	−0.01	0.01	−0.04
Level genetic clinical activity	−0.05	−0.02	0.17**	0.002
Experience (6–10 vs. <6 years)	−0.07	0.01	−0.06	0.14*
Experience (11–15 vs. <6 years)	0.07	0.07	−0.21**	−0.05
Experience (16–20 vs. <6 years)	0.04	−0.002	−0.10	−0.03
Experience (>20 vs. <6 years)	0.11	−0.07	−0.11	−0.04
Specific genetic training (yes)	0.01	0.03	0.10	0.04
Modifying BC risk factor perceived important (reproductive)	0.04	0.08	−0.03	0.02
Modifying BC risk factor perceived important (lifestyle)	0.05	0.14	−0.04	−0.13
Modifying BC risk factor perceived important (hormonal)	−0.04	−0.17**	0.04	0.03
Modifying BC risk factor perceived important (BMI)	0.04	−0.12	−0.05	0.02
Numerical risk communication (absolute figure)	−0.01	−0.19***	0.08	0.11*
	Multiple R^2^ = 0.10*	Multiple R^2^ = 0.15***	Multiple R^2^ = 0.12**	Multiple R^2^ = 0.25***

*BMI* body mass index

^a^log of time to record data in minutes

^b, c, d^higher score = more positive opinion

*p < 0.05; **p < 0.01; ***p < 0.001

Respondents who reported more frequent weekly genetic clinical activity had more positive opinions regarding BWA v3 risk presentations (*p* < 0.01). In this respect, compared to respondents with ‘less than 6 years’ clinical seniority, those with a ‘11 to 15 years’ seniority expressed more negative opinions (*p* < 0.01).

Greater ease of risk communication using BWA v3 was expressed by respondents with intermediate ‘6 to 10 years’ level of seniority compared to those with ‘less than 6 years’ (*p* < 0.05). Numerical (absolute figure) BC risk communication was associated with greater ease of BC risk communication using BWA v3.

Data entry time using BWA v3 was perceived as shorter by men (*p* < 0.01) compared to women. In addition, data entry was perceived as longer by respondents from Australia (*p* < 0.05), France (*p* < 0.05), Germany (*p* < 0.001), and Southern and Western European countries (*p* < 0.01), compared to those from the UK. Compared to the UK, respondents from Australia and the US judged the BWA v3 clinical utility more favourably. Ease of risk communication using BWA v3 was perceived as less positive by French (*p* < 0.001) but more positive by US respondents (*p* < 0.01) compared to UK participants.

## Discussion

The BOADICEA model is being developed further to include all known genetic and non-genetic BC risk factors. In parallel, the BWA is being modified to fulfil more closely clinicians’ and patients’ requirements. In this study we performed an online survey to assess clinicians’ appraisals of BWA v3, and their professional background and practice correlates.

Besides BOADICEA, there are many other BC genetic risk prediction tools available for clinical practice through web-based interfaces. Examples are the BRCAPRO [26, 27] or IBIS [28, 29]. To our knowledge, the use and acceptance of these among clinicians has not yet been systematically studied. Currently, BOADICEA incorporates high and intermediate-high risk mutations, BC pathology, family history of prostate or pancreatic cancer as well as data from relatives of any degree of relatedness [3]. The implementation of comprehensive BC risk assessment, including hormonal, lifestyle and reproductive factors in the near future, should improve its clinical utility substantially.

Survey participants from various countries favourably appraised the scientific validity of the BOADICEA model and BWA v3 risk presentations. Few respondents (9%) expressed that the BOADICEA model was not sufficiently validated. This reflects an awareness, amongst BWA v3 users, of studies that describe the performance of the model in predicting the likelihood of carrying a *BRCA1* or *BRCA2* mutation [30] or the risk of developing breast or ovarian cancer [11, 31] in different populations.

However, a number of respondents (22%) thought that their clinical judgment was as good as or better than BOADICEA risk estimates. Moreover, about half of respondents stated that BOADICEA risk estimates would not change their clinical judgement on breast or ovarian cancer risk management. Less favourable opinions on the clinical utility of BWA v3 may be partly explained by: (1) inconsistencies in clinical guidelines between different BC risk prediction models or timeframes [31]; (2) the fact that BWA v3 does not link computed risks with specific clinical recommendations; or (3) insufficient information provided by the model (e.g., to predict BC risk after prior surgery). On-going extensions to the BOADICEA model and BWA are expected to address these limitations.

Lower perceived clinical utility of BWA v3 was expressed with increasing importance given to hormonal BC factors such as hormone replacement therapy. The current BOADICEA model does not include these factors in contrast to the Gail and IBIS models [3] but on-going extensions will also include known lifestyle and hormonal risk factors. We note that the absence of other BC risk-modifying factors in the BOADICEA model did not affect respondents’ appraisal of its clinical utility which suggests a training requirement with regard to the role of factors, such as those related to lifestyle, affecting BC prevention [32].

Respondents who tended to express risk as numerical figures also perceived BWA v3 clinical utility less positively. It may be that numerical figures (i.e. integers between 0 and 100) provide less latitude for clinical interpretation than broad risk categories expressed as words (i.e., moderate, high, very high).

Sixty-two percent of respondents perceived that the time required for data entry using BWA v3 was too long. This is expected as, compared to other BC risk prediction models, BOADICEA considers additional BC risk factors [3, 33] and can accommodate large families [9] (BWA v3 can accommodate up to 275 family members). We expected that increased frequency of use of BWA v3 could result in shorter data entry times through improved skills, but this was not observed in this study. However, controlling for gender and country of practice, the time for data entry was perceived as longer by genetic counsellors than clinical geneticists. This difference may be related to role sharing between medical and non-medical genetics clinicians.

The time required for data entry using BWA v3 partly reflects the design of the software. BWA v3 captures input pedigree data using HTML forms (form-based data entry). However, it is clear that graphical pedigree data entry programs (that enable users to create a pedigree drawing, with small forms to enter data for each family member) can often capture pedigree data sets more quickly and easily than form-based programs such as BWA v3. The first version of the BWA was released for general use to the healthcare community in 2007. Since that time, advances in client-side software development technologies have facilitated the implementation of Web-based graphical pedigree building tools, and so the BWA will be extended to include such tools in the future. In addition, the time required for data entry using BWA v3 partly reflects the data requirements of the underlying BOADICEA model (e.g., BWA v3 requires that the user specifies a year of birth in order for a family member to be taken into account in a risk calculation, whereas the IBIS tool does not).

Respondents who reported more frequent genetic clinical activity judged BWA v3 risk presentations more positively. However, compared to a clinical experience of less than 6 years, respondents with ‘11 to 15 years’ seniority expressed less positive appraisals of BWA v3 risk presentations. A non-significant trend in this relationship was also revealed for longer seniority.

To be used in clinical practice, a risk assessment tool must present risk estimates in such a way that they are not only easy to understand, but also easy to communicate to patients. Respondents more inclined to communicate risk in numerical format reported that it was easier to communicate risk using BWA v3. Clinicians presenting risk information as numbers rather than words may possibly feel more adequately understood [34].

Respondents with a clinical experience of ‘6 to 10 years’ reported that it was easier to communicate risks using BWA v3 than those who had a clinical experience of less than 6 years. This may be due to the combination of higher than 6 years clinical experience with more recent genetics training than beyond 10 years.

Compared to respondents from the UK, risk communication using BWA v3 was found to be easier for US respondents but more difficult for French users. The effect of country of practice is difficult to explain in this study. Clinicians from the US have reported positive experiences using the BRCA tool [35] with women carrying a *BRCA1* or *BRCA2* mutation [20]. However, in some cultures, the use of direct presentation of cancer risk figures and curves in clinical practice may be found less acceptable as it suggests clinicians’ fear of causing increased counselees’ cancer-specific anxiety. Providing information on cancer risk is complex both cognitively and emotionally. In a recent US survey on communication skills which involved non-genetic clinicians, managing patients’ emotions was found to be mostly difficult [36], which underlines the importance of devoting time for this aspect in cancer genetics training.

### Study limitations and strengths

The study has several limitations. The sample is self-selected based on willingness to participate and over-represents BWA users. NGS from only four countries were solicited; this resulted in a disproportional number of respondents’ from the UK and French NGS compared to other countries. However, the overall sample was large which allowed for multivariate analyses to be performed. Participants varied in age, country of practice and years of experience so a wide range of clinicians provided their opinions.

Although genetics health professionals are currently the main targeted users of the BWA, further survey addressing BWA clinical use should strive to reach non-genetic clinicians such as breast surgeons, oncology specialists and general practitioners who will be increasingly involved in BC risk counselling [37].

## Conclusions

This international survey revealed that the BWA is mostly valued by health professionals’ using it. However, considering that further BOADICEA development plans to include additional factors, to facilitate uptake of the BWA in clinical practice, technological (e.g., step-wise assessment), organisational initiatives (e.g., involving patients or non-genetic professionals such as a nurse navigator [37]) and training initiatives should also be considered. We intend to repeat this survey when the new version of the BWA becomes available to monitor its acceptability.

## Electronic supplementary material

Below is the link to the electronic supplementary material.

Supplementary material 1 (PDF 188 KB)
