# Supplementary material for: Use of the BOADICEA Web Application in clinical practice: appraisals by clinicians from various countries
Source: Fam Cancer. 2017 Jun 16;17(1):31–41. doi: 10.1007/s10689-017-0014-x (PMC5770489; doi:10.1007/s10689-017-0014-x)
Supplement: Supplementary file 1 — Supplementary material 1 (PDF 188 KB) [file 10689_2017_14_MOESM1_ESM.pdf]

Genetic Counselling Practice for Breast and Ovarian Cancer Predisposition

BOADICEA WEB-BASED TOOL SURVEY

- Information document -

We invite you to answer to the following questions as you may provide breast cancer risk counselling in clinical consultations using the BOADICEA web-based tool. Your responses will help us improve the usability of the BOADICEA web-based application.

This survey is carried out within the European project entitled "Breast Cancer Risk after Diagnostic Gene Sequencing (BRIDGES)" led by Prof. Dr. Peter Devilee, Leiden University Medical Centre, and is undertaken by Cologne University in Germany and Curie Institute in France, under the responsibility of Prof. Dr. Rita Schmutlzer, gynaecologist; Prof. Dr. Dominique Stoppa-Lyonnet, clinical geneticist.

The BOADICEA risk prediction model is currently undergoing further development within ongoing research programmes (including BRIDGES) which will provide a BOADICEA^{plus} tool version incorporating new genetic, non-genetic and lifestyle risk factors.

The present survey will be repeated when BOADICEA^{plus} will be available to monitor users' satisfaction with the BOADICEA^{plus} tool.

This survey is anonymous. Data will be stored on a specific website created for the study which will be destroyed once the survey is completed (31th july 2016).

As clinicians from any professional background respond to this survey, you may find some questions to be trivial, nevertheless please answer all of them. **The survey will take about 10-15 minutes to complete**.

Prof, Dr Rita Schmutlzer Prof, Dr Dominique Stoppa-Lyonnet

For further information about this survey, please contact:

Prof Dr Rita Schmutlzer<br><br>Leader of BRIDGES WP5<br><br>Head of the Centre for Familial Breast and Ovarian Cancer<br><br>Cologne University Hospital<br><br>Tel: +49 221 478-98409<br><br>E-Mail: Rita.Schmutzler@uk-koeln.de	Prof Dr Dominique Stoppa-Lyonnet<br><br>Principal Investigator – "*Breast Cancer Genetic Counselling Practice and BOADICEA use Study*"<br><br>Head of the Cancer Genetic Clinic<br><br>Institut Curie<br><br>Tel: + 33 1 44 32 46 97<br><br>Email: Dominique.stoppa-lyonnet@curie.fr	Dr Anne Brédart<br><br>Psycho-Oncology Unit<br><br>Institut Curie<br><br>Tel: + 33 1 44 32 40 33<br><br>Email: anne.bredart@curie.net

Thank you very much for your help.

There are 54 questions in this survey

Part 1

The first part of the survey asks about your practice of genetic counselling and testing for cancer predisposition.

[]

How often do you determine family cancer history in your practice?

Percentage of your clinical time.

*****

Please choose **only one** of the following:

- ◯ <20%
- ◯ 20-40%
- ◯ 40-60%
- ◯ 60-80%
- ◯ >80%

[]How do you obtain a patient's family cancer history? *

Please choose **all** that apply:

- ☐ I draw a family pedigree during the consultation
- ☐ The patient completes a paper questionnaire prior to the consultation
- ☐ The patient submits family history data online prior to the consultation
- ☐ I do not collect patients' family cancer history
- ☐ Other: ____________

[]

To how many patients do you provide risk counselling for breast or ovarian cancer predisposition?

*****

Please choose **only one** of the following:

- ◯ None
- ◯ Less than 5 patients per week
- ◯ 5 to 10 patients per week
- ◯ 11 to 20 patients per week
- ◯ More than 20 patients per week

[]

For which kind of patients?

*

Only answer this question if the following conditions are met:
Answer was ' More than 20 patients per week ' *or* ' 11 to 20 patients per week ' *or* ' 5 to 10 patients per week' *or* ' Less than 5 patients per week' at question '3 [I3a]' (To how many patients do you provide risk counselling for breast or ovarian cancer predisposition?)

Please choose **only one** of the following:

- Only healthy women
- Only affected women
- Both

[]

To how many patients do you provide risk counselling for other cancer predisposition?

*

Please choose **only one** of the following:

- None
- Less than 5 patients per week
- 5 to 10 patients per week
- 11 to 20 patients per week
- More than 20 patients per week

[]For which kind of patients? *

Only answer this question if the following conditions are met:
Answer was 'Less than 5 patients per week' *or* '5 to 10 patients per week' *or* '11 to 20 patients per week' *or* 'More than 20 patients per week' at question '5 [I4a]' (To how many patients do you provide risk counselling for other cancer predisposition?)

Please choose **only one** of the following:

- Only healthy women
- Only affected women
- Both

[]Are you a geneticist / genetic counsellor? *

Please choose **only one** of the following:

- Yes
- No

[]

How many patients do you refer for breast or ovarian cancer predisposition counselling to a geneticist or genetic counsellor ?

*

Only answer this question if the following conditions are met:
Answer was 'No' at question '7 [I5a]' (Are you a geneticist / genetic counsellor?)

Please choose **only one** of the following:

- None
- Less than 5 patients per week
- 5 to 10 patients per week
- 11 to 20 patients per week
- More than 20 patients per week

[]

Which kind of patients do you refer to a genetic specialist?

*

Only answer this question if the following conditions are met:
Answer was 'Less than 5 patients per week' *or* '5 to 10 patients per week' *or* '11 to 20 patients per week' *or* 'More than 20 patients per week' at question '8 [I5b]' (How many patients do you refer for breast or ovarian cancer predisposition counselling to a geneticist or genetic counsellor ?)

Please choose **only one** of the following:

- Only healthy women
- Only affected women
- Both

[]

To how many patients do you order a genetic test in case of cancer predisposition?

*****

Please choose **only one** of the following:

- None
- Less than 5 patients per week
- 5 to 10 patients per week
- 11 to 20 patients per week
- More than 20 patients per week

[]

For which kind of patients?

*****

Only answer this question if the following conditions are met:
Answer was 'Less than 5 patients per week' *or* '5 to 10 patients per week' *or* '11 to 20 patients per week' *or* 'More than 20 patients per week' at question '10 [I6a]' (To how many patients do you order a genetic test in case of cancer predisposition?)

Please choose **only one** of the following:

- Only healthy women
- Only affected women
- Both

[]

To how many patients do you disclose cancer genetic test results?

*****

Please choose **only one** of the following:

- None
- Less than 5 patients per week
- 5 to 10 patients per week
- 11 to 20 patients per week
- More than 20 patients per week

[]

For which kind of patients?

*

Only answer this question if the following conditions are met:
Answer was 'Less than 5 patients per week' *or* '5 to 10 patients per week' *or* '11 to 20 patients per week' *or* 'More than 20 patients per week' at question '12 [I7a]' (To how many patients do you disclose cancer genetic test results?)

Please choose **only one** of the following:

- Only healthy women
- Only affected women
- Both

[]

To how many patients do you order a genetic test for breast cancer treatment decision making?

*

Please choose **only one** of the following:

- None
- Less than 5 patients per week
- 5 to 10 patients per week
- 11 to 20 patients per week
- More than 20 patients per week

[]

To how many patients do you order a genetic test for ovarian cancer treatment decision making?

*

Please choose **only one** of the following:

- None
- Less than 5 patients per week
- 5 to 10 patients per week
- 11 to 20 patients per week
- More than 20 patients per week

[]

Do you have a <u>specifically dedicated</u> consultation for cancer genetic counselling?

*****

Please choose **only one** of the following:

- ◯ Yes
- ◯ No

[]

Do you participate in cancer genetic multidisciplinary team meeting?

*****

Please choose **only one** of the following:

- ◯ Yes
- ◯ No

Part 2

The second part of the survey addresses your utilisation of cancer genetic risk guidelines, prediction models and tools

[]

How often do you consider your national eligibility criteria for referring to breast or ovarian cancer genetic testing ?

Percentage of your clinical time.

*****

Please choose **only one** of the following:

- <20%
- 20-40%
- 40-60%
- 60-80%
- >80%

[]

For you, how important are the following familial and clinical factors to estimate breast cancer risk?

*****

Please choose the appropriate response for each item:

	1. Least important	2.	3.	4.	5. Most important
Family cancer history	○	○	○	○	○
Personal cancer history	○	○	○	○	○
Breast tumour pathology	○	○	○	○	○

[]For you, how important are the following risk modifying factors to estimate breast cancer risk? *

Please choose the appropriate response for each item:

	1. Least important	2.	3.	4.	5. Most important
Age at first menstrual period	○	○	○	○	○
Age at menopause	○	○	○	○	○
Body mass index	○	○	○	○	○
Child bearing at younger age	○	○	○	○	○
Breast feeding	○	○	○	○	○
Alcohol consumption	○	○	○	○	○
Smoking	○	○	○	○	○
Oral contraception	○	○	○	○	○
Hormone replacement therapy	○	○	○	○	○
Physical exercise	○	○	○	○	○

[]To estimate breast cancer risk, how often do you use the following gene mutation or cancer risk prediction models? *

Please choose the appropriate response for each item:

	Never	Occasionally	Regularly	Always	Don't know the model
Manchester scoring system	○	○	○	○	○
Myriad-BRCAtool	○	○	○	○	○
Gail model	○	○	○	○	○
Claus model	○	○	○	○	○
Eisinger score	○	○	○	○	○
BRCAPRO	○	○	○	○	○
IBIS- Tyrer-Cuzick	○	○	○	○	○
BOADICEA	○	○	○	○	○

[]If you use Manchester scoring system, how long, approximately, does it take to record data (minutes) ? *

Only answer this question if the following conditions are met:
Answer was 'Occasionally' *or* 'Regularly' *or* 'Always' at question '21 [II3a]' (To estimate breast cancer risk, how often do you use the following gene mutation or cancer risk prediction models? (Manchester scoring system))

Please write your answer here:

[]

If you use Myriad-BRCAtool, how long, approximately, does it take to record data (minutes) ?

*

Only answer this question if the following conditions are met:
Answer was 'Occasionally' *or* 'Regularly' *or* 'Always' at question '21 [II3a]' (To estimate breast cancer risk, how often do you use the following gene mutation or cancer risk prediction models? (Myriad-BRCAtool))

Please write your answer here:

[]If you use Gail model, how long, approximately, does it take to record data (minutes) ? *

Only answer this question if the following conditions are met:
Answer was 'Occasionally' *or* 'Regularly' *or* 'Always' at question '21 [II3a]' (To estimate breast cancer risk, how often do you use the following gene mutation or cancer risk prediction models? (Gail model))

Please write your answer here:

[]If you use Claus model, how long, approximately, does it take to record data (minutes) ? *

Only answer this question if the following conditions are met:
Answer was 'Occasionally' *or* 'Regularly' *or* 'Always' at question '21 [II3a]' (To estimate breast cancer risk, how often do you use the following gene mutation or cancer risk prediction models? (Claus model))

Please write your answer here:

[]

If you use Eisinger score, how long, approximately, does it take to record data (minutes) ?

*

Only answer this question if the following conditions are met:
Answer was 'Occasionally' *or* 'Regularly' *or* 'Always' at question '21 [II3a]' (To estimate breast cancer risk, how often do you use the following gene mutation or cancer risk prediction models? (Eisinger score))

Please write your answer here:

[]If you use BRCAPRO, how long, approximately, does it take to record data (minutes) ? *

Only answer this question if the following conditions are met:
Answer was 'Occasionally' *or* 'Regularly' *or* 'Always' at question '21 [II3a]' (To estimate breast cancer risk, how often do you use the following gene mutation or cancer risk prediction models? (BRCAPRO))

Please write your answer here:

[]If you use IBIS- Tyrer-Cuzick, how long, approximately, does it take to record data (minutes) ? *

Only answer this question if the following conditions are met:
Answer was 'Occasionally' *or* 'Regularly' *or* 'Always' at question '21 [II3a]' (To estimate breast cancer risk, how often do you use the following gene mutation or cancer risk prediction models? (IBIS- Tyrer-Cuzick))

Please write your answer here:

[]

If you use BOADICEA, how long, approximately, does it take to record data (minutes) ?

*

Only answer this question if the following conditions are met:
Answer was 'Occasionally' *or* 'Regularly' *or* 'Always' at question '21 [II3a]' (To estimate breast cancer risk, how often do you use the following gene mutation or cancer risk prediction models? (BOADICEA))

Please write your answer here:

[]

To estimate breast cancer risk, do you use another gene mutation or cancer risk prediction model?

*

Please choose **only one** of the following:

- Yes
- No

[]Please specify which other model you use

*

Only answer this question if the following conditions are met:
Answer was 'Yes' at question '30 [II4]' (To estimate breast cancer risk, do you use another gene mutation or cancer risk prediction model?)

Please write your answer here:

[]To estimate breast cancer risk, how often do you use the gene mutation or cancer risk prediction model that you indicate previously? *

Only answer this question if the following conditions are met:
Answer was 'Yes' at question '30 [II4]' (To estimate breast cancer risk, do you use another gene mutation or cancer risk prediction model?)

Please choose **only one** of the following:

- Never
- Occasionally
- Regularly
- Always

[]

When you use the gene mutation or cancer risk prediction model that you indicated previously, how long, approximately, does it take to record data (minutes) ?

*

Only answer this question if the following conditions are met:
Answer was 'Yes' at question '30 [II4]' (To estimate breast cancer risk, do you use another gene mutation or cancer risk prediction model?)

Please write your answer here:

[]

To estimate breast cancer risk, do you use still another gene mutation or cancer risk prediction model?

*

Only answer this question if the following conditions are met:
Answer was 'Yes' at question '30 [II4]' (To estimate breast cancer risk, do you use another gene mutation or cancer risk prediction model?)

Please choose **only one** of the following:

- Yes
- No

[]Please specify which other model you use

*

Only answer this question if the following conditions are met:
Answer was 'Yes' at question '34 [II4d]' (To estimate breast cancer risk, do you use still another gene mutation or cancer risk prediction model?)

Please write your answer here:

[]

To estimate breast cancer risk, how often do you use the gene mutation or cancer risk prediction model that you indicate previously?

*

Only answer this question if the following conditions are met:
Answer was 'Yes' at question '34 [II4d]' (To estimate breast cancer risk, do you use still another gene mutation or cancer risk prediction model?)

Please choose **only one** of the following:

- Never
- Occasionally
- Regularly
- Always

[]

When you use the gene mutation or cancer risk prediction model that you indicated previously, how long, approximately, does it take to record data (minutes) ?'

*

Only answer this question if the following conditions are met:
Answer was 'Yes' at question '34 [II4d]' (To estimate breast cancer risk, do you use still another gene mutation or cancer risk prediction model?)

Please write your answer here:

[]How do you communicate these breast or ovarian cancer risks? *

Please choose the appropriate response for each item:

	Never	Occasionally	Regularly	Very often
Relative lifetime risks	○	○	○	○
Absolute lifetime risks	○	○	○	○
Absolute risks for 5, 10, 15 years	○	○	○	○

[]Do you offer psychosocial counselling to support the decision-making process high risk women concerning preventive measures? *

Please choose **only one** of the following:

- ○ Never
- ○ Occasionally
- ○ Regularly
- ○ Always

Part 3

The third part of the survey enquires about the BOADICEA web-based tool

[]What do you think about the BOADICEA Web-based tool usefulness and usability? *

Please choose the appropriate response for each item:

	Strongly agree	Agree	Neither agree nor disagree	Disagree	Strongly disagree
I think that my clinical judgement is as good as or better than the estimates provided by this tool	○	○	○	○	○
I think the tool is not sufficiently scientifically supported or validated for use in my practice	○	○	○	○	○
I think data entry takes too much time using this tool	○	○	○	○	○
I have not enough skills/training to understand the estimates provided by this tool	○	○	○	○	○

[]Please indicate in the space below what changes you would like to see to the BOADICEA web-based tool to make it easier to use (e.g., to enter data, to store data calculations, to produce report, to read report...)

Please write your answer here:

[]What do you think about the BOADICEA Web-based tool usefulness and usability? *

Please choose the appropriate response for each item:

	Strongly agree	Agree	Neither agree nor disagree	Disagree	Strongly disagree
I think the probabilities and percentages provided in the output tables are difficult to understand	○	○	○	○	○
I think the graphs showing risk curves are difficult to understand	○	○	○	○	○
I think the timeframe of the risk estimates is unclear	○	○	○	○	○
I fear of upsetting patients using this tool with them	○	○	○	○	○
I fear that patients misunderstand their risks using this tool with them	○	○	○	○	○

Part 3 suite

How often do the situations below occur in your practice?

[]You think that the patient would be eligible for risk reduction mastectomy <u>but</u> after looking at BOADICEA numerical and graphical results you think that she is not eligible for risk reduction mastectomy anymore *

Please choose **only one** of the following:

- Never
- Occasionally
- Regularly
- Very often

[]You think that the patient would NOT be eligible for risk reduction mastectomy <u>but</u> after looking at BOADICEA numerical and graphical results you think that she is eligible for risk reduction mastectomy *

Please choose **only one** of the following:

- Never
- Occasionally
- Regularly
- Very often

[]You think that the patient would be eligible for risk reduction salpingo-oophorectomy <u>but</u> after looking at BOADICEA numerical and graphical results you think that she is not eligible for risk reduction salpingo-oophorectomy anymore *

Please choose **only one** of the following:

- Never
- Occasionally
- Regularly
- Very often

[]You think that the patient would NOT be eligible for risk reduction salpingo-oophorectomy but after looking at BOADICEA numerical and graphical results you think that she is eligible for risk reduction salpingo-oophorectomy *

Please choose **only one** of the following:

- Never
- Occasionally
- Regularly
- Very often

Part 4

The final section of the survey will ask about you and your clinical practice

[]What is your age? *

Please choose **only one** of the following:

- 20-29
- 30-39
- 40-49
- 50-59
- 60 or above

[]What is your gender? *

Please choose **only one** of the following:

- Female
- Male

[]What is the country of your clinical practice? *

Please write your answer here:

[]You are a
*

Please choose **only one** of the following:

- Clinical geneticist
- Genetic counsellor
- Gynaecologist/obstetricians
- Radiologist
- Pathologist
- Oncology surgeon
- Medical oncologist
- Radiation oncologist
- General practitioner
- Other

[]

During a typical month, approximately what percentage of your professional time do you spend in the following activities?

(Percentage of your time)

*

Please choose the appropriate response for each item:

	<20%	20-40%	40-60%	60-80%	>80%
Patient care					
Research					

[]Since how long do you provide patient care? *

Please choose **only one** of the following:

- 1-5 years
- 6-10 years
- 11-15 years
- 16-20 years
- 21 years or more
- Does not apply

[]Did you receive specific training in cancer genetic testing and counselling? *

Please choose **only one** of the following:

- Yes
- No

[]Please specify which training : *

Only answer this question if the following conditions are met:
Answer was 'Yes' at question '53 [IV7]' (Did you receive specific training in cancer genetic testing and counselling?)

Please write your answer here:

Thank you very much for your help.

Submit your survey.
Thank you for completing this survey.
